# Dynamic changes in DNA methylation and hydroxymethylation revealed the transformation of advanced adenoma into colorectal carcinoma

**DOI:** 10.1002/ctm2.1202

**Published:** 2023-02-28

**Authors:** Yanqi Dang, Ruohui Xu, Jiashu Pan, Xiaoli Xiao, Shengan Zhang, Wenjun Zhou, Yangxian Xu, Guang Ji

**Affiliations:** ^1^ Institute of Digestive Diseases, Longhua Hospital Shanghai University of Traditional Chinese Medicine Shanghai China; ^2^ Department of Digestive Disease, Longhua Hospital Shanghai University of Traditional Chinese Medicine Shanghai China; ^3^ Department of General Surgery, Longhua Hospital Shanghai University of Traditional Chinese Medicine Shanghai China

Dear Editor

Globally, colorectal carcinoma (CRC) ranks third in terms of prevalence according to the latest Global Cancer Statistics.^1^ Studies have shown that most CRCs begin as preexisting adenomas,^2^ among which advanced adenomas (AAs) have been demonstrated to be a more intense risk factor.^3^ Exploring the underlying mechanism of AA‐CRC transformation is helpful in providing a basis for the precise treatment of CRC. DNA 5‐methylcytosine (5mC) and 5‐hydroxymethylcytosine (5hmC) could play major roles in CRC.^4^, ^5^ A single‐tube methylation‐specific quantitative polymerase chain reaction (PCR) assay could be a good predictor of CRC recurrence,^4^ and 5‐hmC levels of zw10 kinetochore protein could have a high diagnostic performance for early‐stage CRC.^5^ However, the functions of 5mC and 5hmC in AA‐CRC transformation remain unclear. Therefore, we conducted an integrated analysis of 5mC and 5hmC to elucidate the mechanism underlying AA‐CRC transformation. Detailed information regarding the study design, participant recruitment and methods was provided in Additional File 1.

First, to verify the role of 5mC in AA‐CRC transformation, 5mC profiles were obtained. Differentially methylated sites (DMSs) and differentially methylated genes (DMGs) were identified (Figure 1A; Figure S1A–C; and Additional File 2). Subsequently, the main biological functions of DMGs were verified, including ubiquitin‐mediated proteolysis, the transforming growth factor‐beta pathway, and pluripotency of stem cells (Figure S1D,E; Additional File 3). Based on the characterization of 5mC in AA‐CRC transformation, the 5mC levels in AA and CRC were further investigated. The results showed that 5mC levels were significantly decreased in AA and then markedly increased in CRC, consistent with the results of sequencing (Figure 1B). Tissue microarrays (TMAs) showed that 5mC levels were also markedly increased in CRC (Figure 1C,D), and patients with CRC with high 5mC levels had a short overall survival (Figure 1E). In addition, 5hmC levels were markedly decreased in CRC tissues (Figure 1F,G). However, no correlation was found between 5hmC levels and the overall survival of patients with CRC (Figure 1H). The levels of 5mC and 5hmC were further verified using immunofluorescence (Figure 1I). These results revealed dynamic changes in 5mC and 5hmC during AA‐CRC transformation.

**FIGURE 1:**
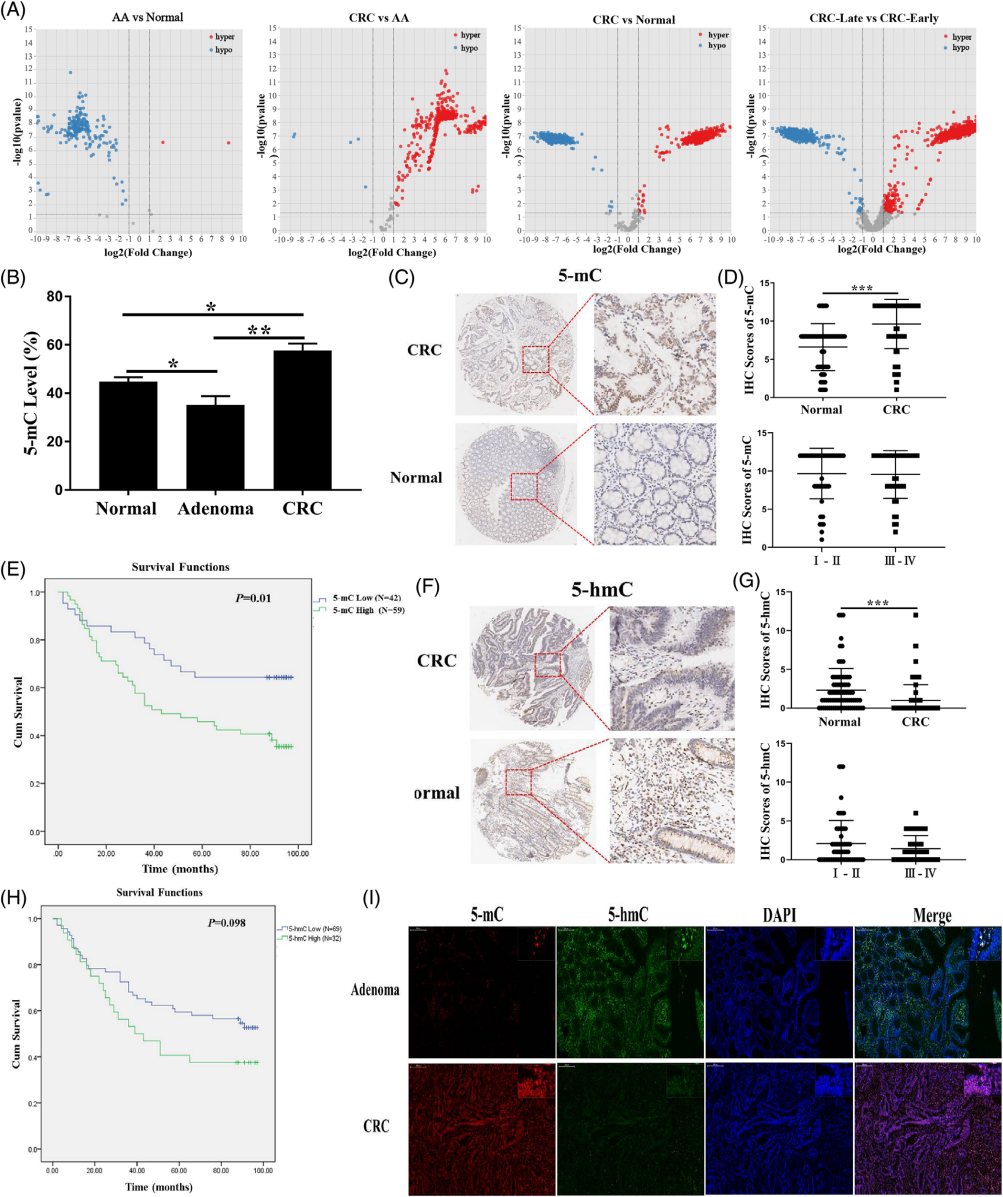
Dynamic changes in 5‐methylcytosine and 5‐hydroxymethylcytosine levels verified in adenoma and colorectal carcinoma tissues. (A) DMSs showed using volcano map in pairwise comparisons. (B) 5mC levels in adenoma and CRC assayed using DNA methylation assay. (C) Representative images of IHC staining for 5mC levels. (D) IHC staining scores of 5mC. (E) Kaplan–Meier overall survival analysis of 5mC levels in patients with CRC. (F) Representative images of IHC staining for 5hmC levels. (G) IHC staining scores of 5hmC. (H) Kaplan–Meier overall survival analysis of 5hmC levels in patients with CRC. (I) 5mC and 5hmC levels in adenoma and CRC assayed through immunofluorescence. Data are presented as mean ± standard deviations. ^*^ *p* < .05; ^**^ *p* < .01; ^***^ *p* < .001. CRC, colorectal carcinoma; 5mC, 5‐methylcytosine; 5hmC, 5‐hydroxymethylcytosine; DMSs, differentially methylated sites; IHC, immunohistochemistry.

5hmC is a stable derivative catalyzed by tet methylcytosine dioxygenases (TETs) in DNA demethylation. To determine dynamic changes in 5mC and 5hmC, we further analyzed 5mC profiles combined with data of 5hmC published in a previous study.^5^ Compared with theAA group, the majority of DMSs in 5mC were hypermethylated in the CRC group (Figure 1A), but the majority of DMSs in 5mC+5hmC were hypermethylated (Figure 2A). Hypermethylated changes in 5mC mainly occurred in the open sea, and changes in 5mC+5hmC occurred in the open sea, N‐shore, S‐shore, N‐shelf, S‐shelf, and CpG island (Figure 2B). Hypermethylated 5mC DMSs were enriched in other and first exon regions (Figure 2C), while hypermethylated changes in 5mC+5hmC were enriched in other, the transcriptional start site 1500 and first exon regions (Figure 2C). In addition, enrichment of hypermethylated changes in 5mC+5hmC in the enhancer was also found (Figure 2D).

**FIGURE 2:**
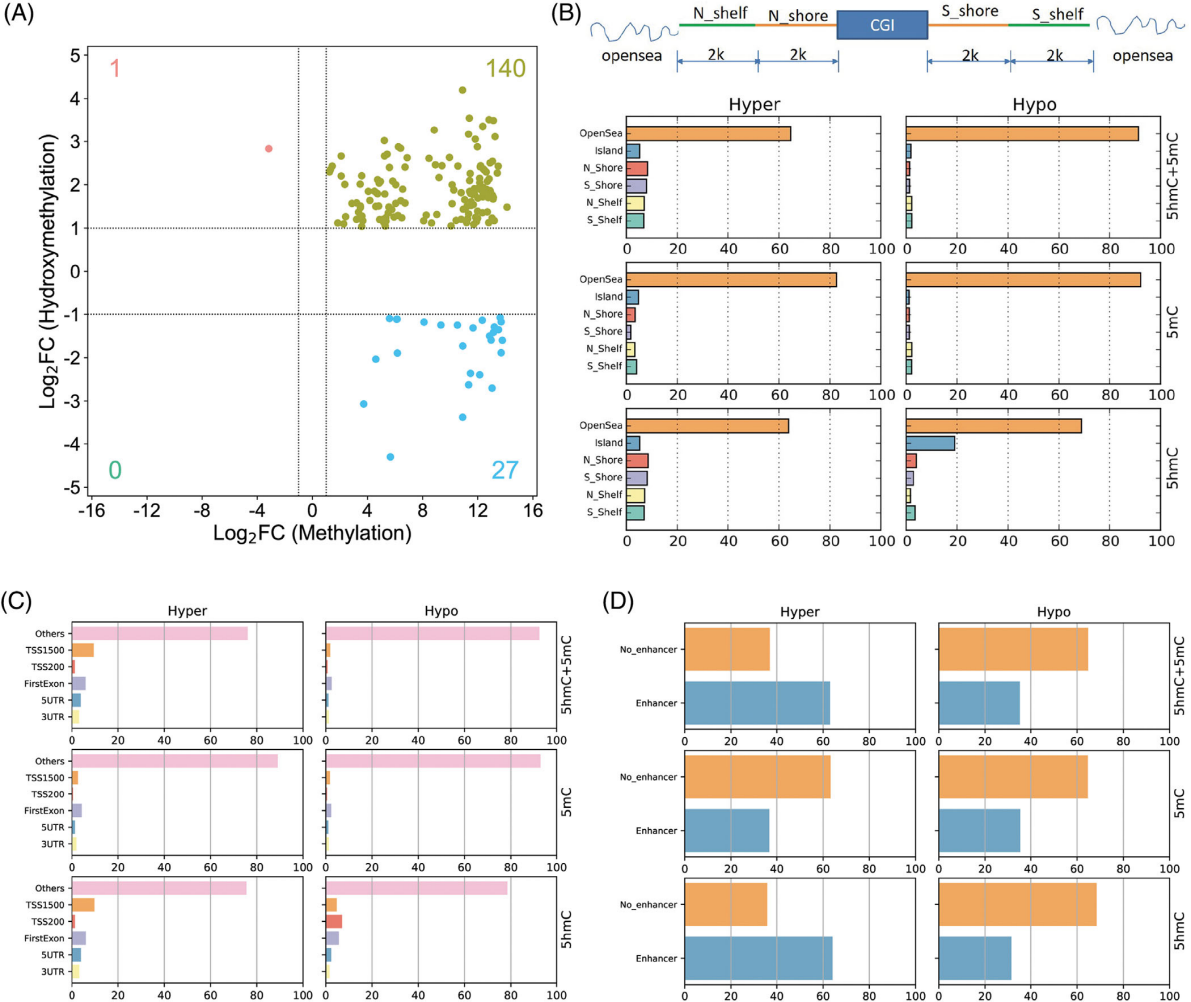
Combined analysis of 5‐methylcytosine and 5‐hydroxymethylcytosine in adenoma and colorectal carcinoma. (A) Relative frequencies of DMSs in 5mC+5hmC, 5mC, and 5hmC; percentage of DMSs in 5mC+5hmC, 5mC, and 5hmC across (B) CGI‐related features, (C) gene features, and (D) enhancers. CGI, CpG island; CRC, colorectal carcinoma; 5mC, 5‐methylcytosine; 5hmC, 5‐hydroxymethylcytosine; DMS, differentially methylated site.

We then performed a combinational analysis of hypermethylated genes and hypohydroxymethylated genes, and 20 overlapped genes were chosen (Figure 3A). The levels of five genes (*ANO10, SUCLG2, PPARGC1A, LRBA*, and *ATP8A1*) were positively correlated with the overall survival of patients with CRC (Figure S2). Moreover, compared with the AA group, mRNA and protein levels of *PPARGC1A, LRBA*, and *ATP8A1* but not *ANO10* and *SUCLG2* were both markedly decreased in the CRC group (Figure 3B–F). The 5hmC levels of *PPARGC1A, LRBA*, and *ATP8A1* were markedly decreased, and the levels of 5mC were significantly higher in the CRC group than in the AA group (Figure 3G–I). Analysis of the SurvivalMeth database showed that the 5mC levels of *PPARGC1A, LRBA*, and *ATP8A1* were negatively correlated with the overall survival of patients with CRC (Figure 3J–L).

**FIGURE 3:**
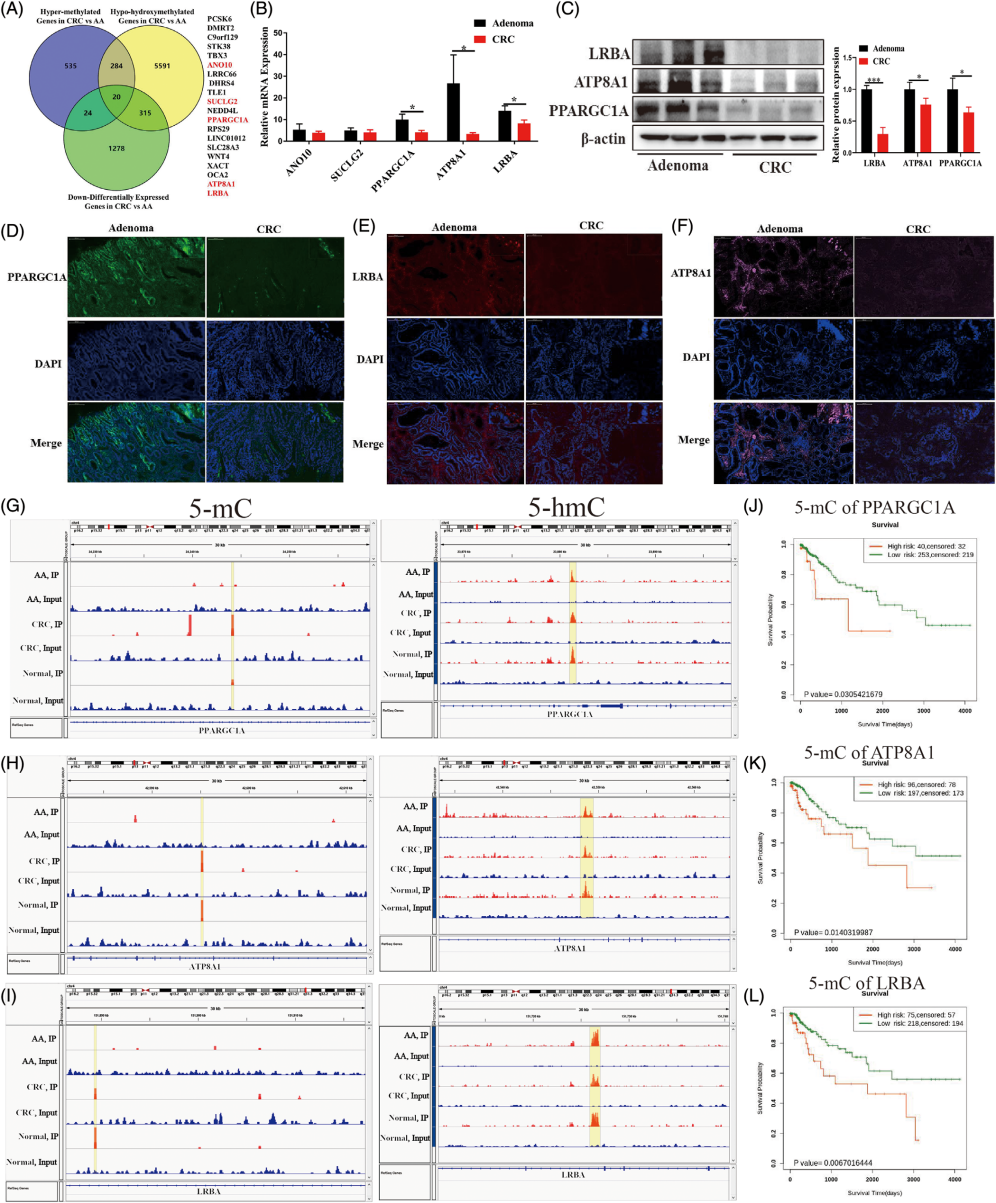
Expression analysis of differentially methylated genes in adenoma and colorectal carcinoma. (A) Venn diagram verifying DMGs between adenoma and CRC; expression of ANO10, SUCLG2, PPARGC1A, LRBA, ATP8A1 in adenoma and CRC verified using (B) RT‐qPCR, (C) western blotting, and (D–F) immunofluorescence; abundances of 5mC and 5hmC in (G) PPARGC1A, (H) ATP8A1, (I) LRBA determined using UCSC Genome Browser; overall survival of patients with methylated levels in (J) PPARGC1A, (K) ATP8A1, (L) LRBA showed using database of SurvivalMeth. Data are presented as mean ± standard deviation. ^*^ *p* < .05; ^***^ *p* < .001. CRC, colorectal carcinoma; DMGs, differentially methylated genes; 5mC, 5‐methylcytosine; 5hmC, 5‐hydroxymethylcytosine; RT‐qPCR, real‐time quantitative polymerase chain reaction.

To elucidate the mechanism of AA‐CRC transformation, the levels of DNA methylases and demethylases were measured. The results showed that DNMT3B levels were significantly increased in AA‐CRC transformation but not DNMT1 and DNMT3A (Figure 4A,B; Figure S3A–C). TET2 levels were significantly decreased in AA‐CRC transformation but not TET1 and TET3 (Figure 4C,D; Figure S3D–F). Studies have indicated that DNMT3B can accelerate the invasion and migration of CRC and promote CRC development,^6^ and TET2 can inhibit CRC progression.^7^ Our results showed that DNMT3B knockdown and TET2 overexpression significantly inhibited cell proliferation, invasion, and migration (Figure 4E–H; Figure S3G–I). Moreover, DNMT3B knockdown increased the mRNA and protein levels of *PPARGC1A* and *LRBA* but not *ATP8A1* (Figure 4I; Figure S4A). TET2 overexpression also promoted the protein level of *PPARGC1A* but reduced *LRBA* and *ATP8A1* protein levels (Figure 4J). Therefore, *PPARGC1A* may be a downstream target of DNMT3B and TET2. PPARGC1A levels in CRC were negatively associated with DNMT3B levels and positively associated with TET2 levels (Figure S4B–E). Studies have shown that PPARGC1A mediates mitochondrial biogenesis and energy metabolism to regulate tumourigenesis in CRC.^8^, ^9^ Our results also showed that PPARGC1A was markedly decreased in CRC, and PPARGC1A overexpression inhibited cell proliferation, invasion, and migration in HCT116 cells (Figure 4K,L; Figure S4F–H). Moreover, *PPARGC1A* expression was positively correlated with activated dendritic cells, memory resting CD4 T cells, and also related to energy metabolism and mitochondrial gene expression (Figure S4I–K; Additional File 5). In addition, compared with the AA group, the 5‐mC level of PPARGC1A was markedly increased and 5‐hmC level of PPARGC1A was markedly decreased in the CRC group (Figure 4 M,N). These results indicated that PPARGC1A mediated by DNMT3B and TET2 could regulate AA‐CRC transformation.^10^

**FIGURE 4:**
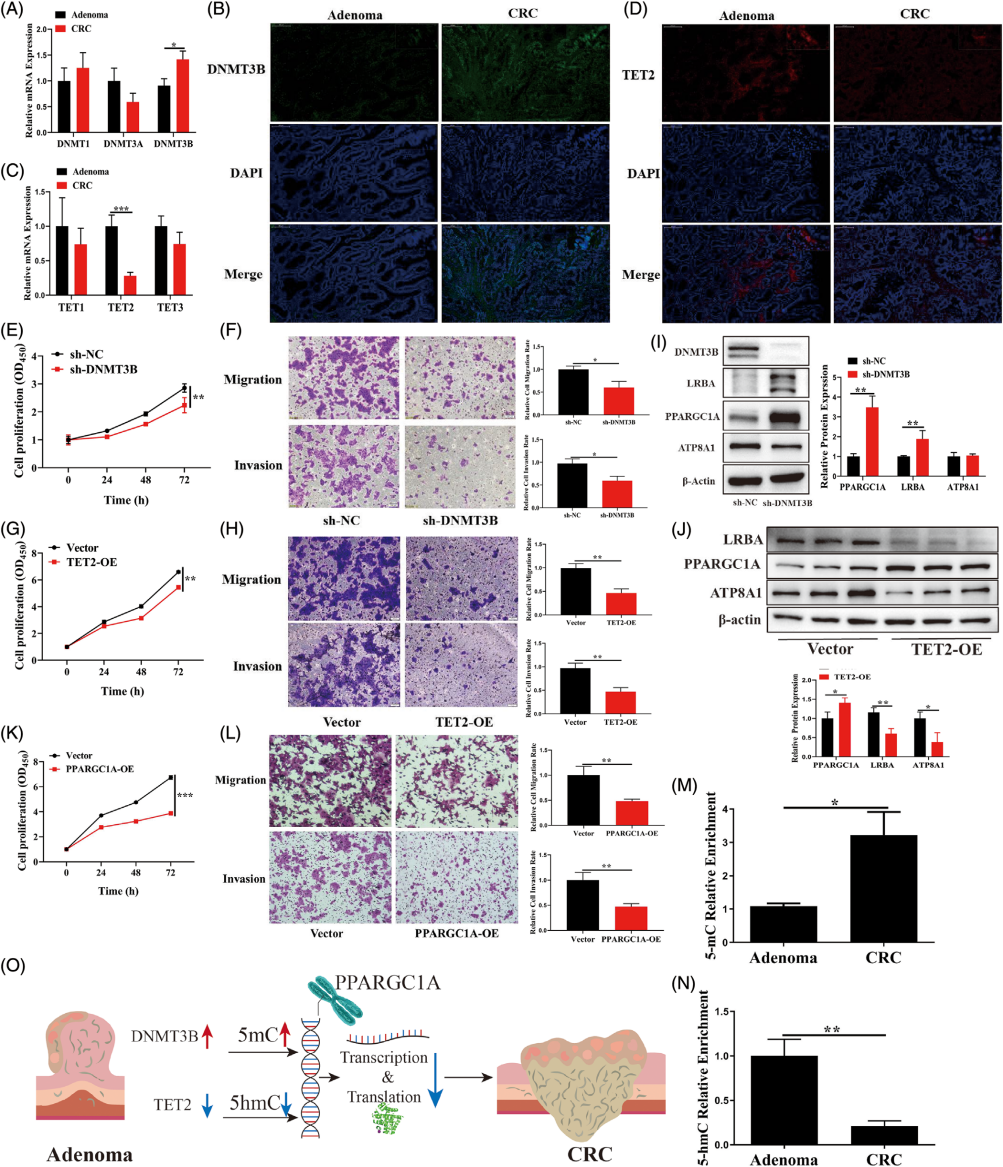
DNMT3B and TET2 regulated colorectal carcinoma progression by mediating 5‐methylcytosine and 5‐hydroxymethylcytosine of *PPARGC1A*. (A and B) DNMT3B expression verified in adenoma and CRC; (C and D) TET2 expression verified in adenoma and CRC; (E) proliferation and (F) transwell assays in HCT116 cells performed after DNMT3B knockdown; (G) proliferation and (H) transwell assays in HCT116 cells performed after TET2 overexpression; (I) protein level of *PPARGC1A* verified after DNMT3B knockdown; (J) protein level of *PPARGC1A* verified after TET2 overexpression; (K) proliferation and (L) transwell assays in HCT116 cells performed after PPARGC1A overexpression; (M and N) 5‐mC and 5‐hmC levels of *PPARGC1A* verified in adenoma and CRC samples; (O) DNMT3B and TET2‐mediated PPARGC1A could regulate the AA‐CRC transformation. Data are presented as mean ± standard deviation. ^*^ *p* < .05; ^**^ *p* < .01; ^***^ *p* < .001. AA, advanced adenoma; CRC, colorectal carcinoma; 5mC, 5‐methylcytosine; 5hmC, 5‐hydroxymethylcytosine.

In summary, 5mC and 5hmC showed dynamic changes in the progression of AA‐CRC transformation. Mechanistically, DNMT3B knockdown and TET2 overexpression inhibited CRC progression. Finally, DNMT3B‐mediated 5mC and TET2‐mediated 5hmC regulated *PPARGC1A* expression, which could regulate the progression of AA‐CRC transformation (Figure 4O). Our results not only suggest critical roles of DNMT3B and TET2 in the AA‐CRC transformation but also provide a new strategy for CRC treatment.

## CONFLICT OF INTEREST STATEMENT

The authors declare no competing interests.

## FUNDING INFORMATION

The Shanghai Rising‐Star Program, Grant Number: 21QA1409000; Shanghai Frontier Research Base of Disease and Syndrome Biology of Inflammatory Cancer Transformation, Grant Number: 2021KJ03–12

## Supporting information

Supporting Information

Supporting Information

Supporting Information

Supporting Information

Supporting Information

Supporting Information

Supporting Information

Supporting Information

Supporting Information

Supporting Information

Supporting Information

Supporting Information
