# Supplementary material for: Dynamic changes in DNA methylation and hydroxymethylation revealed the transformation of advanced adenoma into colorectal carcinoma
Source: Clin Transl Med. 2023 Feb 28;13(3):e1202. doi: 10.1002/ctm2.1202 (PMC9975459; doi:10.1002/ctm2.1202)
Supplement: Supplementary file 1 — Supporting Information [file CTM2-13-e1202-s005.docx]

**Additional file 1**

**Methods**

**Study design**

All the participants were recruited from the department of endoscopy and gastrointestinal surgery. Informed consent was obtained before sample collection. The diagnosis of CRC was confirmed based on pathological evidence. A total of 30 patients with AA and 30 patients with CRC were included. All samples were collected at the Longhua Hospital in Shanghai, China. All specimens were frozen in liquid nitrogen and stored at − 80 °C. This study was approved by the Ethics Committee of Longhua Hospital (2019LCSY020), and informed consent was obtained from all participants.

**Methylated DNA immunoprecipitation sequencing and data analysis**

Methylated DNA immunoprecipitation (MeDIP) sequencing was performed using CloudSeq Biotech Inc. (Shanghai, China). Briefly, genomic DNA was isolated using phenol–chloroform, precipitated with ethanol, and sonicated to 100–500 bp. The sonicated DNA was repaired using the NEBNext® Ultra™ DNA Library Prep Kit. Then, MeDIP was performed with the 5mC antibody (C15200081, Diagenode) according to the manufacturer’s protocol. MeDIP DNA libraries were quantified using an PicoGreen dsDNA Kits (Life Technologies), and subjected to high-throughput 150 base paired-end sequencing on Illumina Hiseq 4000 sequencer according to the manufacturer’s recommended protocol. High quality reads (raw data) were generated after sequencing, image analysis, base calling and quality filtering on Illumina sequencer. cutadapt software was used to for adaptor-trimming, low quality reads removing. After adaptor trimming, the clean reads were aligned to human genome (UCSC HG19) using BOWTIE software (V2.1.0) with default parameters. Peak calling was performed by MACS1.4 software. Differentially methylated sites (DMSs) were identified using the diffReps software. The enriched peaks and DMSs were then annotated with the latest UCSC RefSeq database to connect the peak information with gene annotation. Enriched peaks were visualized using the UCSC Genome Browser.

**DNA methylation assay**

Global DNA methylation levels in adenoma and CRC tissues were detected using the Global DNA Methylation LINE-1 Kit (55017; Active Motif, USA). Briefly, genomic DNA was fragmented by enzymatic digestion and hybridized to a biotinylated human LINE-1 consensus probe. Hybridized DNA was immobilized and incubated with a 5mC antibody and secondary antibody to detect methylated fragments. Colorimetric values were measured spectrophotometrically at 450 nm.

**Immunohistochemistry**

Tissue microarrays (TMAs) were obtained from Shanghai Outdo Biotech Co., Ltd. (Shanghai, China), and immunohistochemistry was performed. Briefly, the TMAs were heated and subjected to antigen retrieval. Endogenous peroxidase activity was blocked using hydrogen peroxide. Finally, the sections were incubated with an anti-5mC antibody (ab10805, Abcam, USA) and anti-5hmC antibody (ab106918, Abcam, USA) and stained with 3, 3'-diaminobenzidine for 3 min. Staining scores were determined according to a previous study [1]. The scores for staining intensity were determined according to a staining intensity scale that ranged from 0 to 3+ points (0 for no staining, 1+ for weak immunoreactivity, 2+ for moderate immunoreactivity, and 3+ for strong immunoreactivity). Positive percentage was scored as follows: 0 for negative cells, 1+ for 1%–25%, 2+ for 26%–50%, 3+ for 51%–75%, and 4+ for 76%–100%. The intensity and positive proportion scores were then multiplied to obtain a composite score, which recorded as the scores of IHC. The composite score ranged from 0 to 12; a below average score indicates low expression, whereas an above average score indicates high expression.

**Immunofluorescence**

5mC antibody (ab10805, Abcam, USA), 5hmC antibody (ab106918, Abcam, USA), PPARGC1A antibody (SC-518025, SANTA, USA), LRBA antibody (NBP1-90765, Novus Biologicals, USA), ATP8A1 antibody (NBP2-30403, Novus Biologicals, USA), DNMT3B (57868, Cell Signaling Technology, USA), and TET2 (GTX124205, GeneTex, USA) were used for immunofluorescence. Briefly, samples were incubated with antibodies overnight at 4 °C. Subsequently, secondary antibodies were incubated for 1 h at 37 °C. Finally, the samples were stained, and images were captured.

**Real-time quantitative polymerase chain reaction**

Clinical tissues and cultured cells were homogenized in TRIzol reagent (Ambion, USA) and then reverse-transcribed into cDNA using the Evo M-MLV reverse transcription kit (Agbio, China). Primers were designed and synthesized (Generay Biotech, Shanghai, China) and are shown in Additional File 6. Real-time quantitative PCR (RT-qPCR) was performed using the SYBR-Green qPCR kit (Applied Biosystems, USA). β-actin served as the reference gene for mRNA expression, and the relative expression of target genes to β-actin was calculated using the 2^−ΔΔT^ method.

**Western blotting**

Proteins were extracted and quantified using RIPA lysis buffer (Beyotime Biotechnology, Shanghai, China) and transferred to a polyvinylidene fluoride membrane. The membranes were blocked for 1 h in 5% fat-free milk at room temperature. Finally, the membrane was incubated with primary and then horseradish peroxidase-conjugated secondary antibodies, including DNMT3B (57868, Cell Signaling Technology, USA), LRBA (NBP1-90765, Novus Biologicals, USA), PPARGC1A (SC-518025, SANTA, USA), ATP8A1 (NBP2-30403, Novus Biologicals, USA), and β-actin (4970s, Cell Signaling Technology). A chemiluminescent substrate was added, and images were captured to calculate the density using ImageJ.

**Cell culture and treatment**

HCT116 and HEK-293T cells were purchased from Shanghai Cell Bank (Shanghai, China) and were cultured in Dulbecco’s Modified Eagle Medium and Roswell Park Memorial Institute 1640 medium containing 10% fetal bovine serum and penicillin/streptomycin (100 U/mL) in an incubator with 5% CO_2_ at 37 °C. DNMT3B short hairpin RNA plasmid (shRNA-DNMT3B, Genomeditech, Shanghai, China) or negative control and TET2 overexpression plasmid (TET2-OE, Generay Biotech, Shanghai, China) PPARGC1A overexpression plasmid (PPARGC1A-OE, Genomeditech, Shanghai, China), or vector plasmids were transfected using the FuGENE® HD kit (Promega, USA) according to previous studies [1, 2].

**Cell counting kit 8 assay**

HCT116 cells were transfected and seeded in 96-well plates at a density of 2 × 10^4^ cells/well and cultured for 0, 24, 48, and 72 h. The supernatant was discarded, and medium containing 10 μL cell counting kit-8 (TargetMol, Shanghai, China) was added to each well and incubated at 37 ℃ for 1 h. The optical density value was measured at 450 nm using a Hybrid 4 microplate reader (BioTek, USA).

**Cell migration and invasion assay**

The migration and invasion abilities of HCT116 cells were assessed. In brief, transfected HCT116 cells were seeded in six-well plates at a concentration of 10^5^ cells and cultured for 48 h. The cells were fixed using 4% paraformaldehyde for 30 min and stained with 0.1% crystal violet solution for 15 min. Five fields under a microscope were randomly selected to calculate the migration and invasion abilities of HCT116 cells.

**Analysis of the SurvivalMeth database**

Correlation in 5mC levels of PPARGC1A, LRBA, ATP8A1 and the overall survival of patients with CRC were analyzed using SurvivalMeth database (http://bio-bigdata.hrbmu.edu.cn/survivalmeth/). A below average score indicates low expression, whereas an above average score indicates high expression.

**MeDIP-qPCR and hMeDIP-qPCR**

The 5mC and 5hmC levels of PPARGC1A were determined using EpiQuik™ Methylated DNA Immunoprecipitation Kit (P-2019, Epigentek Group Inc.) and EpiQuik™ Hydroxymethylated DNA Immunoprecipitation (hMeDIP) Kit (P-1038, Epigentek Group Inc.). The enriched DNA was analyzed through qPCR, and the 5mC and 5hmC enrichment was normalized using input. The primers are listed in Additional File 6: Table S2.

**Statistical analysis**

Data were presented as the mean ± standard deviation and were analyzed using a two-tailed Student’s t-test in SPSS Statistics v20.0. The Kaplan–Meier method was used to generate survival curves, which were compared using the log-rank test. Univariate and multivariate Cox regression analyses were used to analyze survival data. The distribution differences of the variables were analyzed using Pearson’s chi-squared test. P < 0.05 was considered statistically significant.

**References：**

1. Pan J, Liu F, Xiao X, Xu R, Dai L, Zhu M, Xu H, Xu Y, Zhao A, Zhou W, et al: **METTL3 promotes colorectal carcinoma progression by regulating the m6A-CRB3-Hippo axis.** *J Exp Clin Cancer Res* 2022, **41:**19.

2. Dang Y, Hu D, Xu J, Li C, Tang Y, Yang Z, Liu Y, Zhou W, Zhang L, Xu H, et al: **Comprehensive analysis of 5-hydroxymethylcytosine in zw10 kinetochore protein as a promising biomarker for screening and diagnosis of early colorectal cancer.** *Clin Transl Med* 2020, **10:**e125.
