# Supplementary material for: Dynamic changes in DNA methylation and hydroxymethylation revealed the transformation of advanced adenoma into colorectal carcinoma
Source: Clin Transl Med. 2023 Feb 28;13(3):e1202. doi: 10.1002/ctm2.1202 (PMC9975459; doi:10.1002/ctm2.1202)
Supplement: Supplementary file 6 — Supporting Information [file CTM2-13-e1202-s011.docx]

**Additional file 6**

Table S1. Primers sequences of mRNAs in Real-time PCR experiments.

| RNAs | Primer(5'->3') |
| --- | --- |
| ANO10 | Forward: CGTCTGGCATCGTGATTCAG  Reverse: GCAAACCGAGTGTACCAGGT |
| SUCLG2 | Forward: CAAAAGACCCTAATGTTGTGGGA  Reverse: TTCAGCAACCATCACCTTGTT |
| PPARGC1A | Forward: TCTGAGTCTGTATGGAGTGACAT  Reverse: CCAAGTCGTTCACATCTAGTTCA |
| ATP8A1 | Forward: CCCCAAGCCATGTGCTACATT  Reverse: CCTCATCAAACTGTCAACGTCT |
| LRBA | Forward: ATACCCCAGCCAAGGTTCAAC  Reverse: GCACTGTTCCAACTCTCCGAAT |
| DNMT1 | Forward: CACGAAAGCCACCACCACCAAG  Reverse: CTGACACACCTCACAGACGCCA |
| DNMT3A | Forward: CTCTCTTTGATGGAATCGCTAC  Reverse: ACTCCTGGATATGCTTCTGTGT |
| DNMT3B | Forward: GCAAAGACCGAGGGGATGAA  Reverse: CTGCCACAAGACAAACAGCC |
| TET1 | Forward: CTCTTGGTATGAGTGGGAGTGTTGT  Reverse: GTTTGATGATGATTGAGGAGTGTGT |
| TET2 | Forward: CGTAGAGAAGCAGAAGGAAGCAAGA  Reverse: ACAGGAGCAAAGGCAAGTAAACAAT |
| TET3 | Forward: GCAGTTTGAGGCTGAATTTGGAG  Reverse: TTGCTTGGGGGAACGGGTAG |
| β-actin | Forward: GAAGAGCTACGAGCTGCCTGA  Reverse: CAGACAGCACTGTGTTGGCG |

Table S2. Primers sequences of MeDIP-qPCR

| RNAs | Primer(5'->3') |
| --- | --- |
| PPARGC1A | Forward: GAAGAATGGCATGAACC  Reverse: GGAGTCTCGCTCTGTCG |
