# Supplementary figures and images for: Dynamic changes in DNA methylation and hydroxymethylation revealed the transformation of advanced adenoma into colorectal carcinoma
Source: Clin Transl Med. 2023 Feb 28;13(3):e1202. doi: 10.1002/ctm2.1202 (PMC9975459; doi:10.1002/ctm2.1202)

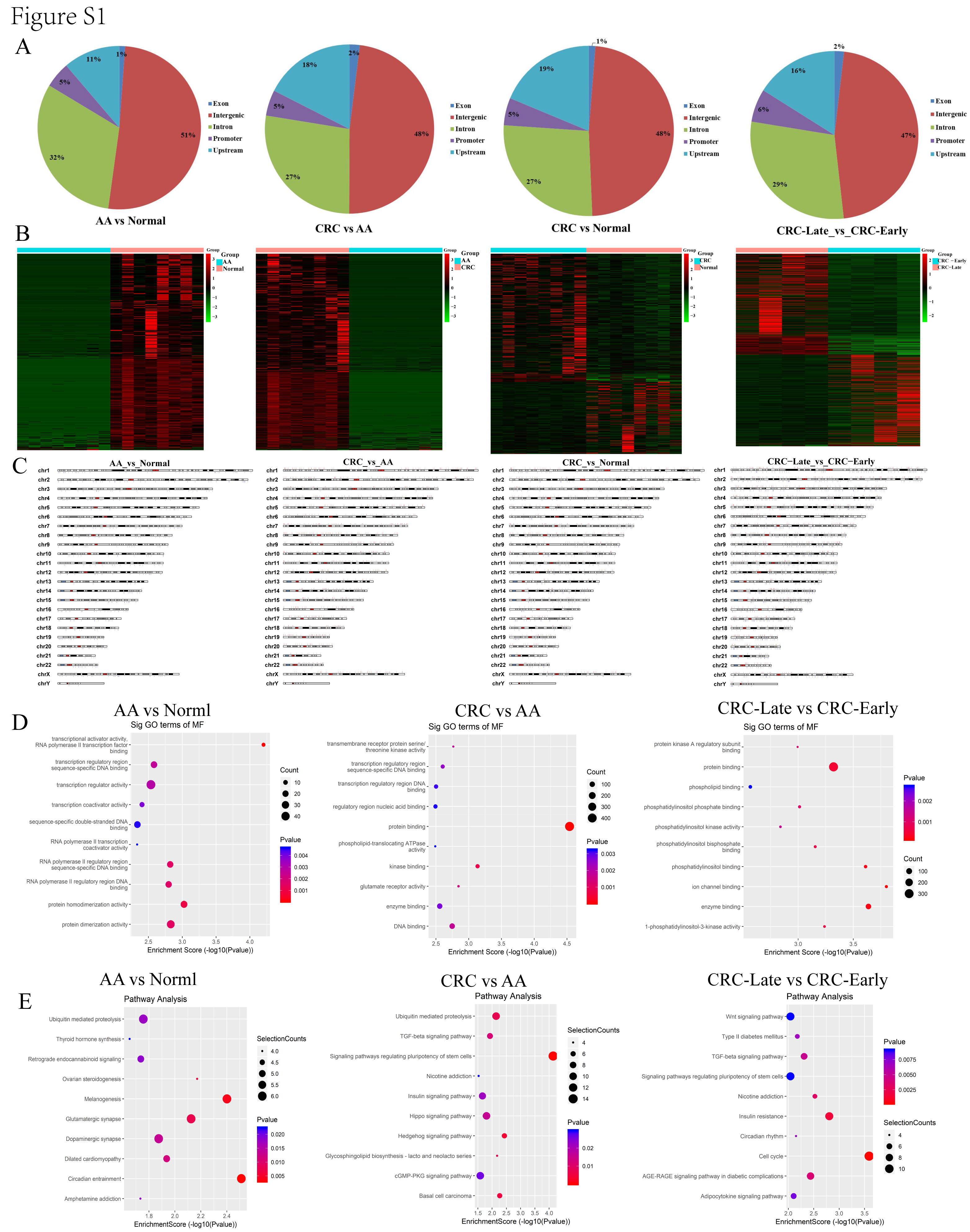

Supplement: Supplementary file 7 — Supporting Information [file CTM2-13-e1202-s001.tif]

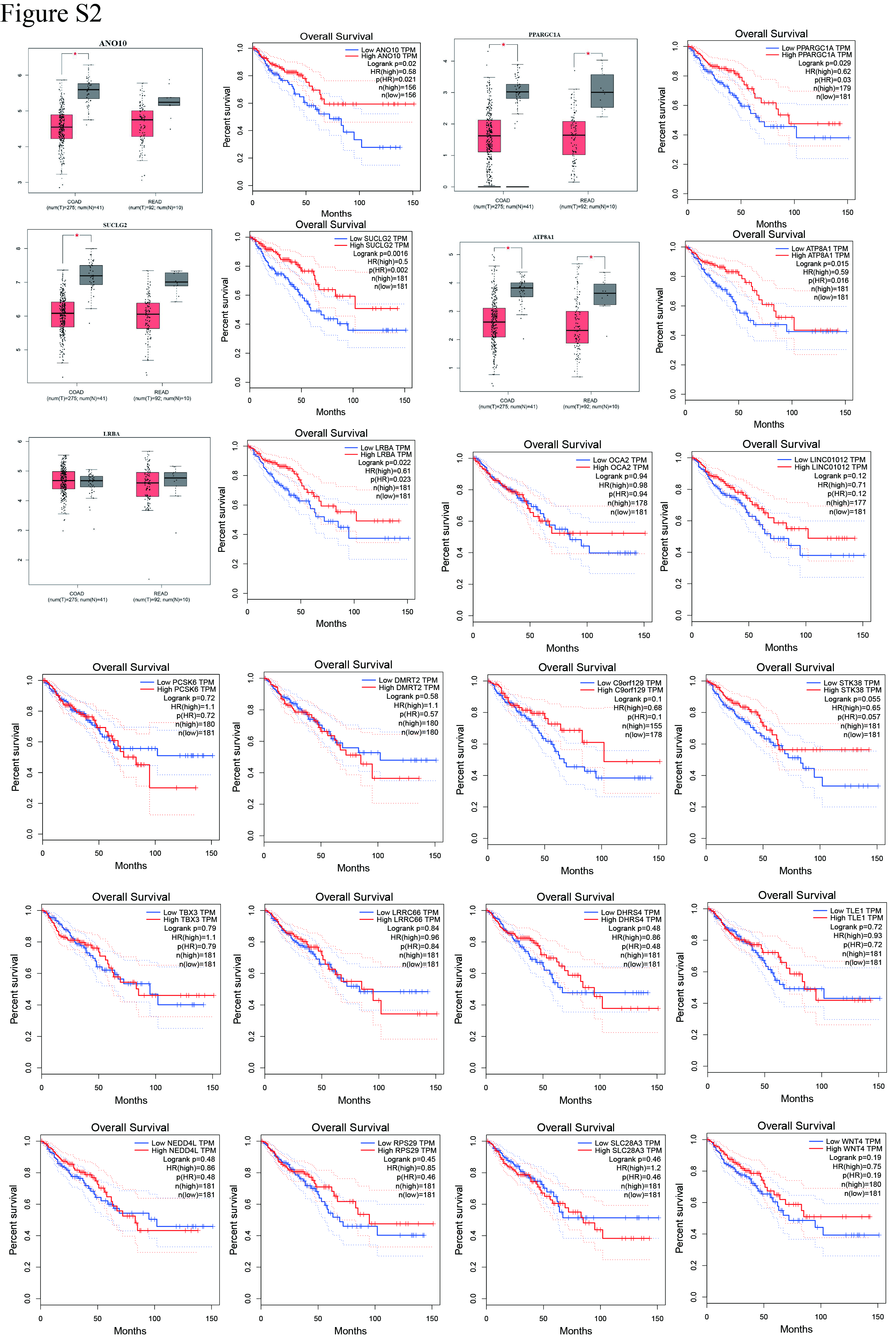

Supplement: Supplementary file 8 — Supporting Information [file CTM2-13-e1202-s003.tif]

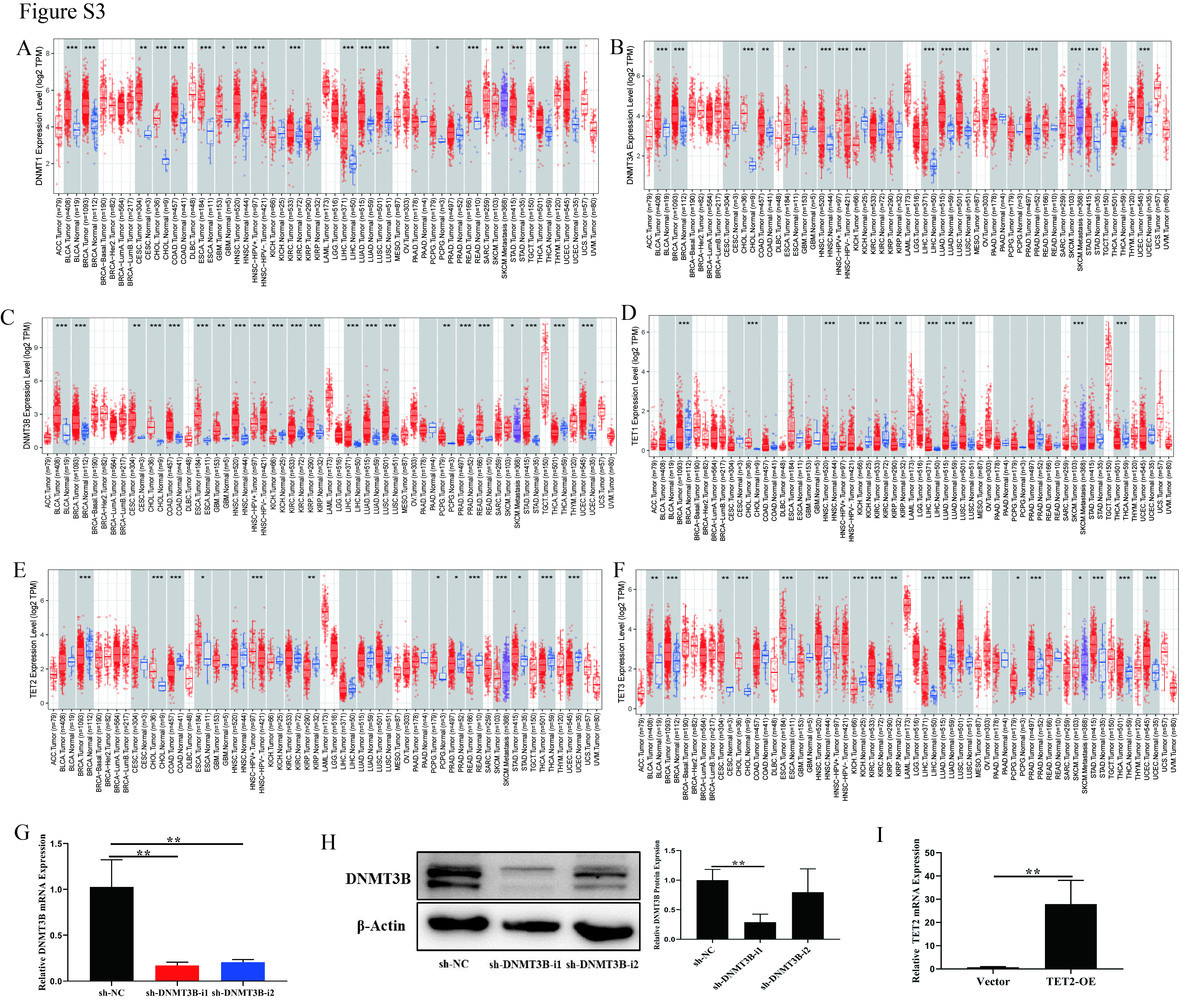

Supplement: Supplementary file 9 — Supporting Information [file CTM2-13-e1202-s007.tif]

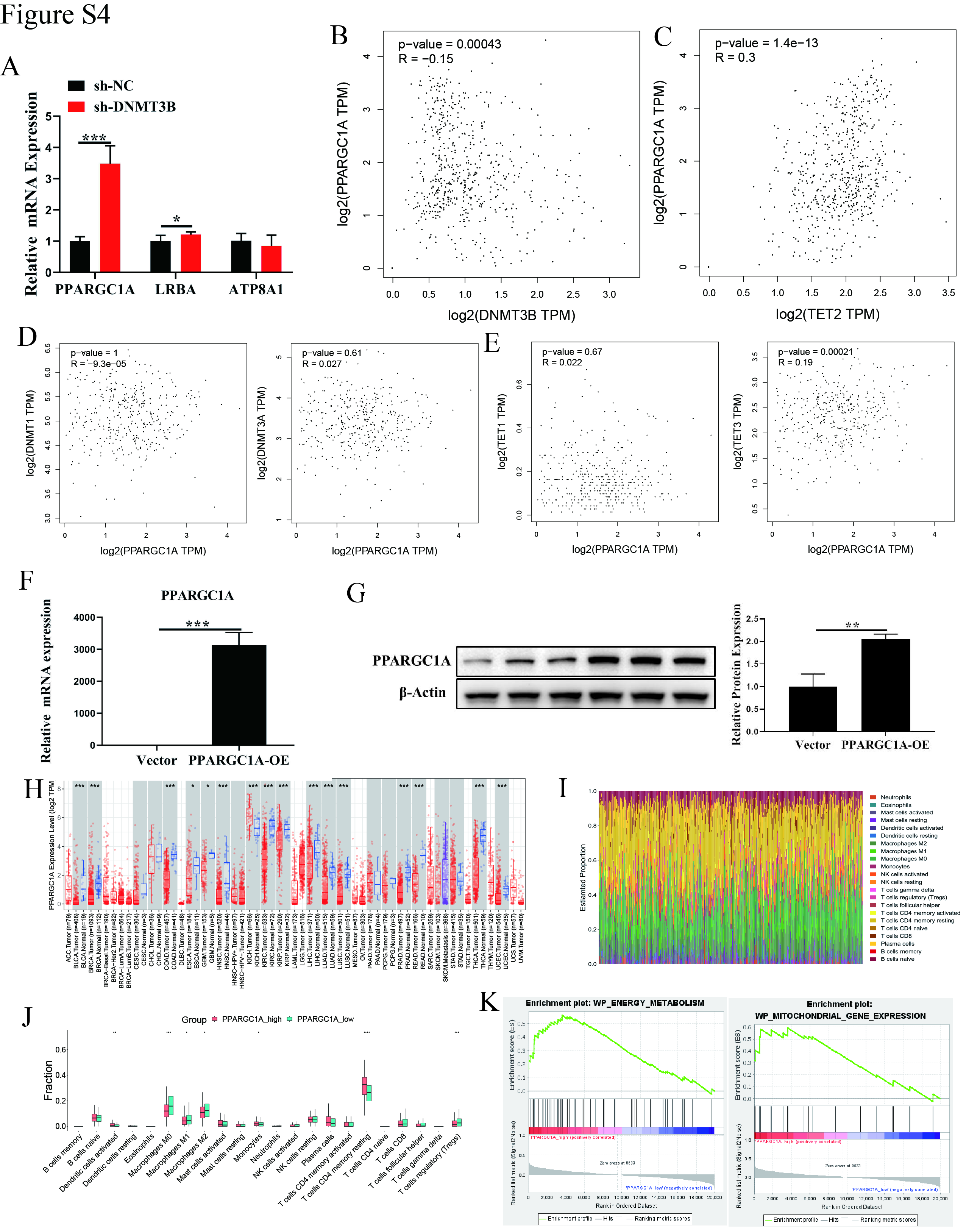

Supplement: Supplementary file 10 — Supporting Information [file CTM2-13-e1202-s002.tif]

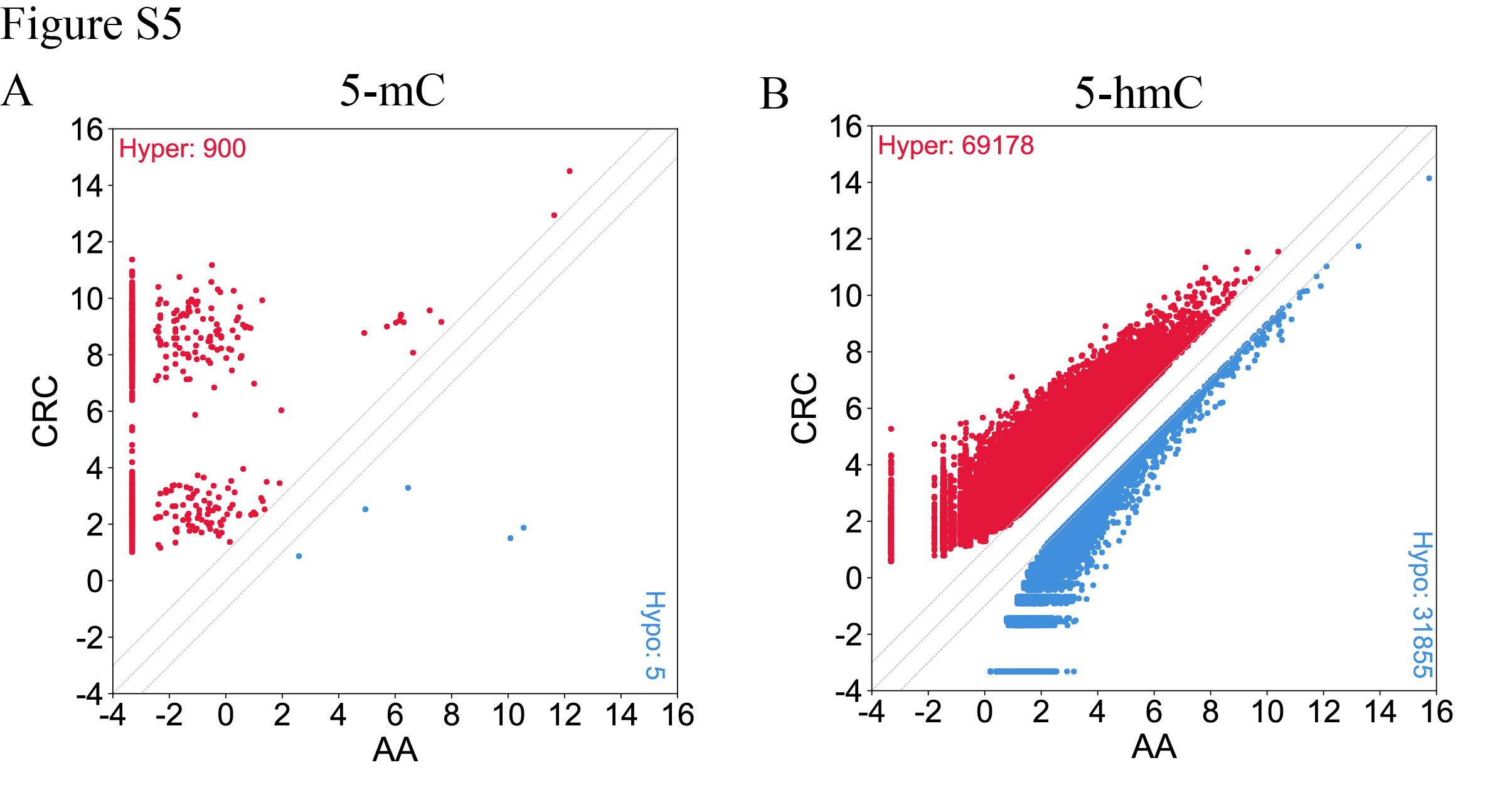

Supplement: Supplementary file 11 — Supporting Information [file CTM2-13-e1202-s008.tif]

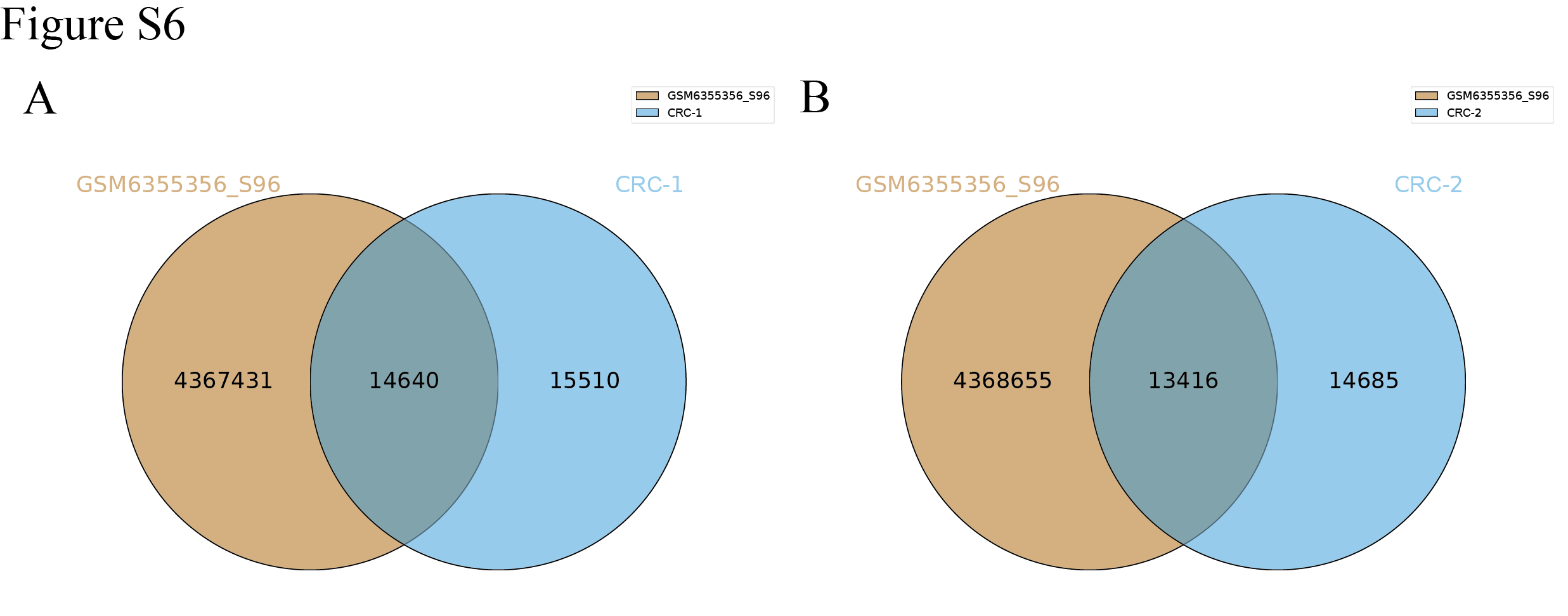

Supplement: Supplementary file 12 — Supporting Information [file CTM2-13-e1202-s012.tif]
